# Negative-pressure-assisted ventilation lowers driving pressure and mechanical power in an ARDS model

**DOI:** 10.1152/japplphysiol.00486.2022

**Published:** 2022-10-13

**Authors:** Elizabeth C. Rohrs, Thiago G. Bassi, Michelle Nicholas, Jessica Wittmann, Marlena Ornowska, Karl C. Fernandez, Matt Gani, Steven C. Reynolds

**Affiliations:** ^1^Fraser Health Authority, Royal Columbian Hospital, New Westminster, British Columbia, Canada; ^2^Lungpacer Medical USA, Inc., Exton, Pennsylvania; ^3^Department of Biomedical Physiology and Kinesiology, Simon Fraser University, Burnaby, British Columbia, Canada

**Keywords:** ARDS, diaphragm neurostimulation, mechanical ventilation, phrenic nerve pacing, ventilator-induced lung injury

## Abstract

Increased lung heterogeneity from regional alveolar collapse drives ventilator-induced lung injury in patients with acute respiratory distress syndrome (ARDS). New methods of preventing this injury require study. Our study objective was to determine whether the combination of temporary transvenous diaphragm neurostimulation (TTDN) with standard-of-care volume-control mode ventilation changes lung mechanics, reducing ventilator-induced lung injury risk in a preclinical ARDS model. Moderate ARDS was induced using oleic acid administered into the pulmonary artery in pigs, which were ventilated for 12 h postinjury using volume-control mode at 8 mL/kg, positive end-expiratory pressure (PEEP) 5 cmH_2_O, with respiratory rate and $\text{F}{\text{I}}_{{\text{O}}_{2}}$ set to achieve normal arterial blood gases. Two groups received TTDN, either every second breath [mechanical ventilation (MV) + TTDN50%, *n* = 6] or every breath (MV + TTDN100%, *n* = 6). A third group received volume-control ventilation only (MV, *n* = 6). At study-end, $\text{P}{\text{a}}_{{\text{O}}_{2}}$/$\text{F}{\text{I}}_{{\text{O}}_{2}}$ was highest and alveolar-arterial oxygen (A-a) gradient was lowest for MV + TTDN100% (*P* < 0.05). MV + TTDN100% had the smallest end-expiratory lung volume loss and lowest extravascular lung water at study-end (*P* < 0.05). Static lung compliance was highest and transpulmonary driving pressure was lowest at baseline, postinjury, and study-end in MV + TTDN100% (*P* < 0.05). The total exposure to transpulmonary driving pressure, mechanical power, and mechanical work was the lowest in MV + TTDN100% (*P* < 0.05). Lung injury score and total inflammatory cytokine concentration in lung tissue were the lowest in MV + TTDN100% (*P* < 0.05). Volume-control ventilation plus transvenous diaphragm neurostimulation on every breath improved $\text{P}{\text{a}}_{{\text{O}}_{2}}$/$\text{F}{\text{I}}_{{\text{O}}_{2}}$, A-a gradient, and alveolar homogeneity, as well as reduced driving pressure, mechanical power, and mechanical work, and resulted in lower lung injury scores and tissue cytokine concentrations in a preclinical ARDS model.

**NEW & NOTEWORTHY** Combining temporary transvenous diaphragm neurostimulation with volume-control ventilation on every breath, called negative-pressure-assisted ventilation, improved gas exchange and alveolar homogeneity in a preclinical model of moderate ARDS. Transpulmonary driving pressure, mechanical power, and mechanical work reductions were observed and resulted in lower lung injury scores and tissue cytokine concentrations in the every-breath-neurostimulation group compared with volume-control ventilation only. Negative-pressure-assisted ventilation is an exciting new potential tool to reduce ventilator-induced lung injury in patients with ARDS.

## Graphical abstract

**Figure:**
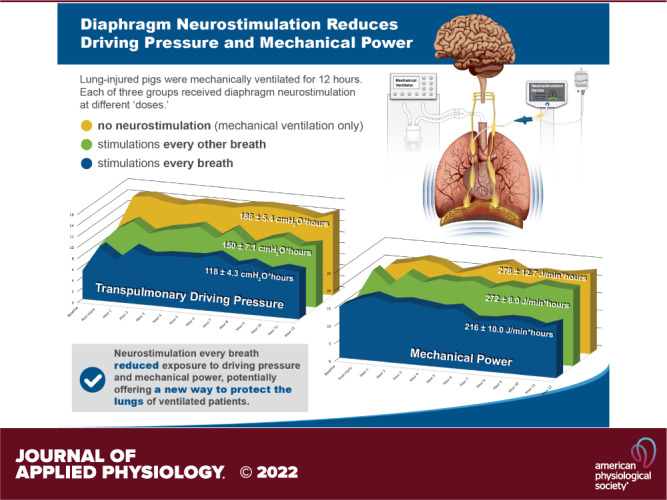

## INTRODUCTION

Acute respiratory distress syndrome (ARDS) drives morbidity and mortality in the intensive care unit (ICU) and is associated with iatrogenic mechanical ventilator-induced lung injury (VILI) (1–3). ARDS has a mortality rate of 35%–46% and is characterized by the rapid onset of severe hypoxic respiratory failure and alterations in pulmonary mechanics such as reduced lung volumes and compliance (4). Current practice aims to prevent VILI in patients with ARDS by limiting ventilator-delivered tidal volume, and emerging practice recommends reducing plateau pressures and driving pressures, and minimizing mechanical power (5–8). Increased transpulmonary plateau and driving pressures, as well as mechanical power, are correlated with injurious lung strain (7, 9–12). Reducing driving pressure and mechanical power can be achieved by improving and sustaining alveolar recruitment and homogeneity thereby improving lung compliance by decreasing lung tissue elastance, minimizing tidal volume, or decreasing extrathoracic pressures (7, 9–12).

The diaphragm is responsible for pleural and transpulmonary pressure generation and its contraction also serves to counteract the upward pressure from abdominal contents onto the lungs, reducing pressure on dependent, dorsal alveoli (13, 14). Maintaining diaphragm contractions by facilitating spontaneous breathing while mechanically ventilated is an effective way to sustain dependent alveolar recruitment, thereby improving alveolar compliance and homogeneity, and reducing driving pressure and mechanical power (15, 16). However, concerns exist about potential injury due to strong spontaneous respiratory efforts in these patients (17, 18). This concern, along with a therapeutic need to provide sedation to effectively care for these patients, means that alternative methods of supporting alveolar recruitment, such as personalizing and optimizing positive end-expiratory pressure (PEEP) are used (16, 19). Negative-pressure ventilation modalities such as the body box, the continuous negative abdominal pressure device, and phrenic-nerve pacing use negative pressure to reduce the risk of VILI posed by mechanical ventilation (20–22).

Our group has previously investigated the use of a novel technology using temporary transvenous diaphragm neurostimulation (TTDN) to stimulate diaphragm contractions synchronously during mechanical-ventilator-delivered breaths (23–26). TTDN generates negative pressure by contracting the diaphragm during inspiration to offset the harmful effects of positive pressure delivered from the ventilator (23–26). We have termed this negative-pressure-assisted ventilation. TTDN resulted in reduced atelectasis formation, improved $\text{P}{\text{a}}_{{\text{O}}_{2}}$/$\text{F}{\text{I}}_{{\text{O}}_{2}}$ and alveolar homogeneity, and reduced driving pressure in a healthy-lung preclinical model ventilated for 50 h (23). As this was in a normal-lung model it limited the generalizability of the findings. Negative-pressure-assisted ventilation may be particularly beneficial in addressing the regional alveolar collapse that drives lung heterogeneity in ARDS (27, 28). This study investigates the impact of TTDN on lung mechanics, dynamics, and injury in a preclinical ARDS model, ventilated for 12 h post-oleic acid-induced lung injury.

## METHODS

Detailed protocol descriptions are provided in the Supplemental Material (see, https://doi.org/10.6084/m9.figshare.20514132).

### Animals

Eighteen female Yorkshire pigs with oleic acid-induced lung injury were ventilated for 12 h postinjury, under ICU conditions. Animals were randomly assigned to one of three groups (*n* = 6 in each): mechanical ventilation only (MV), mechanical ventilation with TTDN on every second breath (MV + TTDN50%), and mechanical ventilation with TTDN on every breath (MV + TTDN100%). A group of six never-intubated, never-ventilated animals were euthanized without receiving intubation or mechanical ventilation and were used for lung histology controls (NV). The study was conducted in accordance with the Canadian Council for Animal Care guidelines, and approved by the University of British Columbia and Simon Fraser University Animal Ethics Committees.

### Lung Injury Protocol

Animals were intubated and sedated with intravenous anesthesia to ablate respiratory drive and ventilated in the supine position using volume-control ventilation (8 mL/kg, PEEP 5 cmH_2_O; 5). Lung injury was induced by administrating *cis*-9-octadecenoic acid (oleic acid) via a pulmonary artery catheter, a validated preclinical ARDS model (29, 30). Oleic acid (0.1–0.4 mL) was mixed with 2 mL of the animal’s arterial blood, and injected into the distal port of the pulmonary artery catheter every 2 min until a $\text{P}{\text{a}}_{{\text{O}}_{2}}$/$\text{F}{\text{I}}_{{\text{O}}_{2}}$ ≤ 200 was achieved (30). Animals were ventilated in a volume-control mode for 12 h postinjury. Only changes to respiratory rate or $\text{F}{\text{I}}_{{\text{O}}_{2}}$ to maintain normal arterial blood gases were permitted. Animals were euthanized at study termination for tissue samples.

### TTDN Protocol

TTDN was delivered via a catheter inserted percutaneously into the left subclavian vein (LIVE Catheter, Lungpacer Medical; 23, 24, 26). Neurostimulation was delivered in synchrony with the inspiratory phase of ventilator-delivered breaths as previously published (23, 24, 26). Neurostimulation was synchronized to the start of inspiration and was set to match inspiration duration (23, 24, 26). TTDN was delivered at 25 Hz with a stimulation intensity targeting a reduction of ventilator mechanical work [pressure-time product (PTP)] of 15%–20% (23, 24, 26).

### Measurements

Transpulmonary pressure, static compliance, and PTP were measured via a respiratory monitor (MBMed) attached to a nasogastric catheter with esophageal pressure monitoring capability (Nutrivent). End-expiratory lung volume loss was measured using electrical impedance tomography (EIT; Dräger). Extravascular lung water (EVLW) was measured using PiCCO thermodilution (Getinge). Mechanical power was calculated as follows: MP = 0.098 × RR × Vt × (peak inspiratory pressure–½ driving pressure) (31).

Lung tissue samples were taken from five standardized areas of the left lung. Alveolar expansion at the time of tissue fixation was measured from blinded samples using alveolar chord length (32). Lung tissue injury was assessed by modifying a standardized histology score as previously published (23, 32). This system scored the number of neutrophils in both the alveolar and interstitial space, and proteinaceous debris filling the airspaces. The score was modified to omit the presence of hyaline membranes and alveolar septal thickening measurements. This is due to the presence of smooth muscle fibers in pig lung tissue. Lungs were not inflated to a standardized transpulmonary pressure postmortem, to avoid postmortem alveolar recruitment. Twenty random fields were selected for scoring, and the independent variables were weighted and normalized to the number of fields evaluated (32). The injury score is a continuous value between 0 (lowest) and 1 (highest; 32).

Lung tissue samples from study-end were flash frozen and subsequently homogenized for protein extraction. Blood was taken at study-end and serum was separated via centrifugation. Cytokine concentrations were measured in both homogenized tissue and serum using porcine-specific ELISA discovery assays.

### Statistical Analysis

A power calculation (α 0.05, β 0.80) based on $\text{P}{\text{a}}_{{\text{O}}_{2}}$/$\text{F}{\text{I}}_{{\text{O}}_{2}}$ changes seen by Grasso et al. (33), negative-pressure ventilation in lung-injured rabbits (350 mmHg vs. 75 mmHg, SD 75), yields two animals per arm. Six per group were studied, to ensure adequate statistical power. Statistical analysis was performed using GraphPad Prism 9.2.0 (GraphPad). Measurements are reported as median (IQR) and compared using nonparametric Kruskal–Wallis ANOVA. Post hoc Dunn’s test of multiple comparisons was performed if the Kruskal–Wallis test showed significance. Longitudinal data were analyzed using a mixed-effects model. This mixed model uses a compound symmetry covariance matrix and is fit using restricted maximum likelihood (REML). Fixed effects were time, TTDN dose [0% (MV), 50% (TTDN on every second breath), 100% (TTDN on every breath)], and TTDN dose interaction with time. The random effect was considered the subjects. A Geisser–Greenhouse correction was made for lack of sphericity. Correlation was measured using nonparametric Spearman correlation analysis.

## RESULTS

### Group Comparisons

The MV, MV + TTDN50%, and MV + TTDN100% groups were not significantly different in weight, fluid balance, sedative drug dose, and oleic acid dose (Table 1). Arterial blood gas measurements of pH, $\text{P}{\text{a}}_{\text{C}{\text{O}}_{2}}$, and $\text{P}{\text{a}}_{{\text{O}}_{2}}$ were not significantly different between groups (Table 1). Tidal volume (8 mL/kg) and PEEP (5 cmH_2_O) were set to be equal for all groups. Median respiratory rate was highest in MV + TTDN100% (*P* = 0.003, Table 1). Median $\text{F}{\text{I}}_{{\text{O}}_{2}}$ was lowest in MV + TTDN100% (*P* = 0.027, Table 1).

**Table 1.** Group comparisons of mechanical ventilator settings, arterial blood gas values, drug dose, weight, fluid balance, and lung injury score

	Median (IQR)			Kruskal–Wallis (*P* Value)	Dunn’s Multiple Comparisons Post Hoc Test	
	MV (*n* = 6)	MV + TTDN50% (*n* = 6)	MV + TTDN100% (*n* = 6)		Group	*P* Value
Respiratory rate, breath/min	20 (20–21)	20 (20–20)	21 (21–22)	0.003	MV vs. MV + TTDN50%	ns
					MV vs. MV + TTDN100%	**0.037**
					MV + TTDN50% vs. MV + TTDN100%	**0.021**
$\text{F}{\text{I}}_{{\text{O}}_{2}}$	0.40 (0.37–0.42)	0.33 (0.33–0.36)	0.32 (0.32–0.47)	0.027	MV vs. MV + TTDN50%	ns
					MV vs. MV + TTDN100%	ns (0.056)
					MV + TTDN50% vs. MV + TTDN100%	ns
pH	7.44 (7.42–7.47)	7.44 (7.39–7.45)	7.44 (7.38–7.46)	ns	MV vs. MV + TTDN50%	
					MV vs. MV + TTDN100%	
					MV + TTDN50% vs. MV + TTDN100%	
$\text{P}{\text{a}}_{\text{C}{\text{O}}_{2}}$, mmHg	52 (47–54)	50 (47–55)	48 (46–54)	ns	MV vs. MV + TTDN50%	
					MV vs. MV + TTDN100%	
					MV + TTDN50% vs. MV + TTDN100%	
$\text{P}{\text{a}}_{{\text{O}}_{2}}$, mmHg	141 (92–152)	128 (110–152)	121 (111–147)	ns	MV vs. MV + TTDN50%	
					MV vs. MV + TTDN100%	
					MV + TTDN50% vs. MV + TTDN100%	
Propofol, mg/kg/h	50 (48–55)	56 (51–58)	56 (48–58)	ns (0.059)	MV vs. MV + TTDN50%	
					MV vs. MV + TTDN100%	
					MV + TTDN50% vs. MV + TTDN100%	
Midazolam, mg/kg/h	70 (68–74)	70 (68–72)	69 (67–73)	ns	MV vs. MV + TTDN50%	
					MV vs. MV + TTDN100%	
					MV + TTDN50% vs. MV + TTDN100%	
Fentanyl, µg/kg/h	390 (360–410)	414 (385–436)	405 (398–423)	ns	MV vs. MV + TTDN50%	
					MV vs. MV + TTDN100%	
					MV + TTDN50% vs. MV + TTDN100%	
Ketamine, mg	1,300 (1,200–2,050)	1,400 (950–1,700)	1,450 (1,175–2,025)	ns	MV vs. MV + TTDN50%	
					MV vs. MV + TTDN100%	
					MV + TTDN50% vs. MV + TTDN100%	
Levophed, µg/min	2.8 (2.4–3.1)	2.8 (2.5–3.0)	2.4 (2.3–2.6)	ns	MV vs. MV + TTDN50%	
					MV vs. MV + TTDN100%	
					MV + TTDN50% vs. MV + TTDN100%	
Phenylephrine, µg	1,200 (1,175–1,600)	1,400 (1,175–1,600)	1,400 (1,200–1,650)	ns	MV vs. MV + TTDN50%	
					MV vs. MV+TTDN100%	
					MV + TTDN50% vs. MV + TTDN100%	
Oleic acid, mL/kg	0.12 (0.07–0.16)	0.11 (0.08–0.20)	0.09 (0.06–0.12)	ns	MV vs. MV + TTDN50%	
					MV vs. MV + TTDN100%	
					MV + TTDN50% vs. MV + TTDN100%	
Weight, kg	64.1 (62.0–69.2)	67.5 (64.3–84.1)	68.8 (66.5–72.7)	ns	MV vs. MV + TTDN50%	
					MV vs. MV + TTDN100%	
					MV + TTDN50% vs. MV + TTDN100%	
Fluid balance, mL/kg/h	1.3 (1.2–1.4)	1.5 (1.3–1.5)	1.3 (1.3–1.3)	ns	MV vs. MV + TTDN50%	
					MV vs. MV + TTDN100%	
					MV + TTDN50% vs. MV + TTDN100%	
Modified lung injury score	0.47 (0.40–0.49)	0.56 (0.48–0.62)	0.39 (0.37–0.39)	0.002	MV vs. MV + TTDN50%	ns
					MV vs. MV + TTDN100%	**0.041**
					MV + TTDN50% vs. MV + TTDN100%	**0.004**

MV, mechanical ventilation; ns, not significant; TTDN, temporary transvenous diaphragm neurostimulation. Bold values are significant *P* values.

### Lung Mechanics, Dynamics, and Function

**Figure 1.**
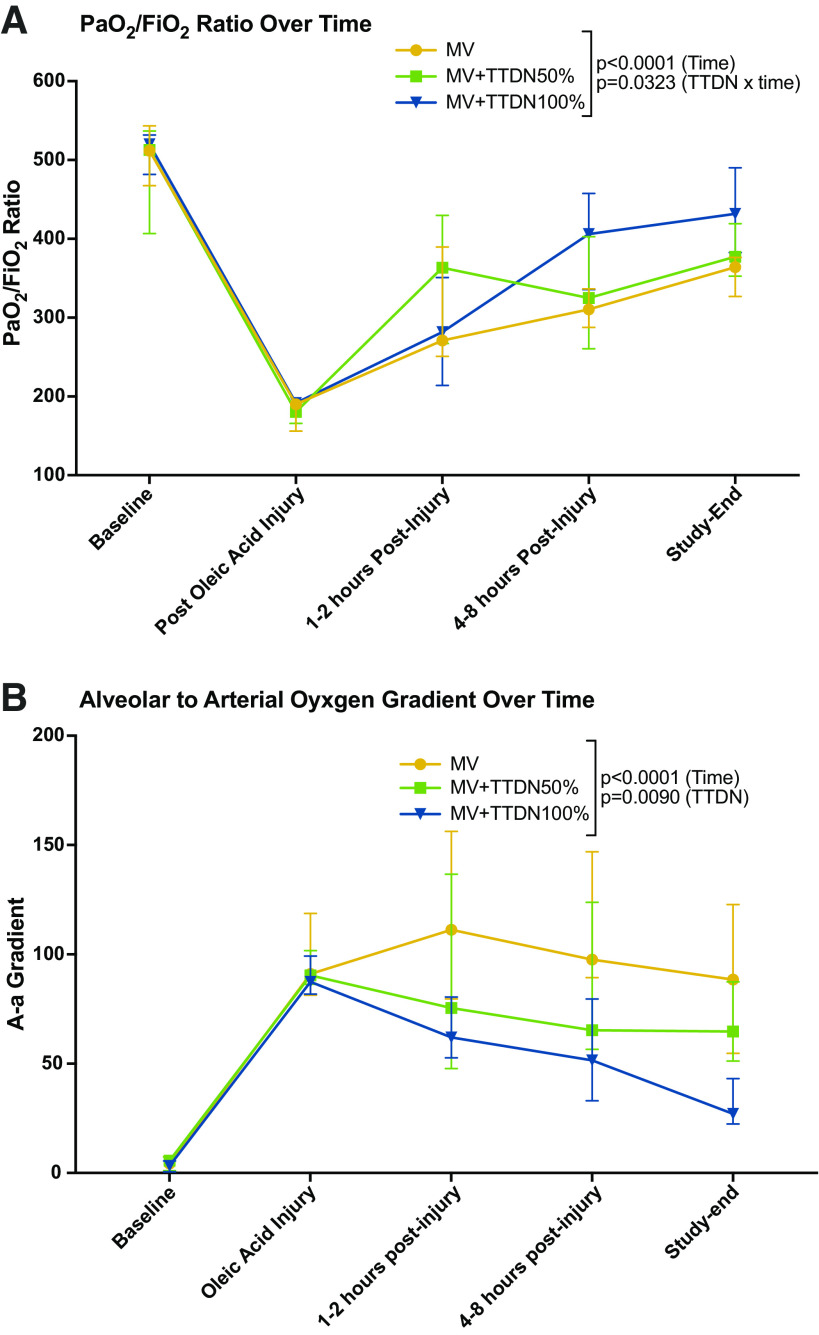
*A*: $\text{P}{\text{a}}_{{\text{O}}_{2}}$/$\text{F}{\text{I}}_{{\text{O}}_{2}}$ over time was significantly affected by temporary transvenous diaphragm neurostimulation (TTDN), and best recovery was seen in the mechanical ventilation (MV) + TTDN100% group (*n* = 6 for all groups, mixed-methods analysis). *B*: alveolar to arterial oxygen gradient over time was significantly affected by TTDN, and best recovery was seen in the MV + TTDN100% group (*n* = 6 for all groups, mixed-methods analysis).

$\text{P}{\text{a}}_{{\text{O}}_{2}}$/$\text{F}{\text{I}}_{{\text{O}}_{2}}$ was not statistically different between groups at baseline or postinjury and was highest in MV + TTDN100% at study-end (*P* = 0.036, Table 2). $\text{P}{\text{a}}_{{\text{O}}_{2}}$/$\text{F}{\text{I}}_{{\text{O}}_{2}}$ over time was affected by time (*P* < 0.001), TTDN dose (*P* = 0.024), and interaction of TTDN dose and time (*P* = 0.032, Table 2 and Fig. 1*A*). Alveolar-arterial oxygen (A-a) gradient was not statistically different between groups at baseline or postinjury, and was lowest in MV + TTDN100% at study-end (*P* = 0.007, Table 2). A-a gradient over time was affected by time (*P* < 0.001) and TTDN dose (*P* = 0.009). The effect of interaction of TTDN dose with time showed a trend to significance (*P* = 0.064, Table 2, Fig. 1*B*).

**Table 2.** $\text{P}{\text{a}}_{{\text{O}}_{2}}$/$\text{F}{\text{I}}_{{\text{O}}_{2}}$, alveolar-arterial oxygen gradient, end-expiratory lung volume change, and extravascular lung water measured at baseline, postinjury, and study-end

	Median (IQR)			Kruskal–Wallis (*P* Value)	Dunn’s Multiple Comparisons Post Hoc Test		Mixed Methods Analysis (*P* Value)		
	MV (*n* = 6)	MV + TTDN50% (*n* = 6)	MV+ TTDN100% (*n* = 6)		Groups	*P* Value	Time	TTDN	TTDN × Time
$\text{P}{\text{a}}_{{\text{O}}_{2}}$/$\text{F}{\text{I}}_{{\text{O}}_{2}}$									
Baseline	512 (467–543)	513 (406–537)	520 (482–532)	ns	MV vs. MV + TTDN50%		**<0.001**	**0.024**	**0.032**
					MV vs. MV + TTDN100%				
					MV + TTDN50% vs. MV + TTDN100%				
Injury	190 (156–197)	180 (166–193)	192 (187–194)	ns	MV vs. MV + TTDN50%				
					MV vs. MV + TTDN100%				
					MV + TTDN50% vs. MV + TTDN100%				
Study-end	364 (327–376)	363 (267–430)	432 (383–490)	**0.036**	MV vs. MV + TTDN50%	ns			
					MV vs. MV + TTDN100%	**0.039**			
					MV + TTDN50% vs. MV + TTDN100%	ns			
Alveolar-arterial oxygen gradient, mmHg									
Baseline	4 (3–8)	6 (1–7)	3 (1–5)	ns	MV vs. MV + TTDN50%		**<0.001**	**0.009**	ns
					MV vs. MV + TTDN100%				
					MV + TTDN50% vs. MV + TTDN100%				
Injury	91 (81–119)	90 (86–102)	87 (82–99)	ns	MV vs. MV + TTDN50%				
					MV vs. MV + TTDN100%				
					MV + TTDN50% vs. MV + TTDN100%				
Study-end	88 (55–123)	65 (51–87)	27 (22–43)	**0.007**	MV vs. MV + TTDN50%	ns			
					MV vs. MV + TTDN100%	**0.011**			
					MV + TTDN50% vs. MV + TTDN100%	ns			
Change (Loss) in end-expiratory lung volume, mL/kg									
Baseline	n/a	n/a	n/a	n/a	MV vs. MV + TTDN50%		**<0.001**	ns	ns
					MV vs. MV + TTDN100%				
					MV + TTDN50% vs. MV + TTDN100%				
Injury	10 (7–14)	12 (5–14)	5 (4–8)	ns (0.058)	MV vs. MV + TTDN50%				
					MV vs. MV + TTDN100%				
					MV + TTDN50% vs. MV + TTDN100%				
Study-end	14 (8–17)	15 (12–18)	8 (5–9)	**0.007**	MV vs. MV + TTDN50%	ns			
					MV vs. MV + TTDN100%	ns			
					MV + TTDN50% vs. MV + TTDN100%	**0.013**			
Extravascular lung water, mL/kg									
Baseline	6 (6–8)	7 (7–8)	6 (5–6)	**0.010**	MV vs. MV + TTDN50%	ns	**<0.001**	ns	ns
					MV vs. MV + TTDN100%	ns			
					MV + TTDN50% vs. MV + TTDN100%	**0.013**			
Injury	9 (8–12)	10 (8–13)	9 (8–11)	ns	MV vs. MV + TTDN50%				
					MV vs. MV + TTDN100%				
					MV + TTDN50% vs. MV + TTDN100%				
Study-end	10 (9–10)	10 (9–13)	8 (7–9)	**0.027**	MV vs. MV + TTDN50%	ns			
					MV vs. MV + TTDN100%	ns			
					MV + TTDN50% vs. MV + TTDN100%	**0.039**			

Change over time was compared using mixed-methods analysis. MV, mechanical ventilation; ns, not significant; TTDN, temporary transvenous diaphragm neurostimulation. Bold values are significant *P* values.

**Figure 2.**
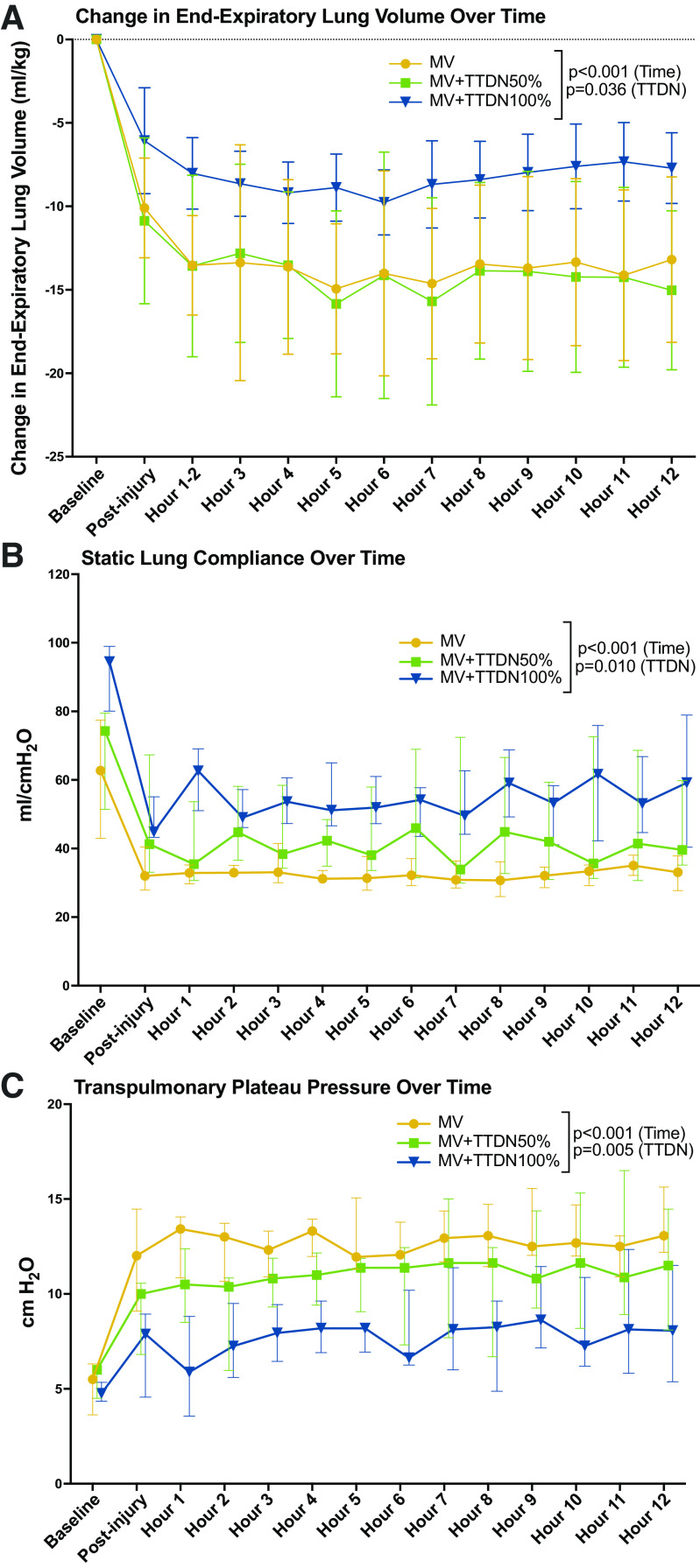
*A*: change in end-expiratory lung volume over time was significantly affected by temporary transvenous diaphragm neurostimulation (TTDN), and the mechanical ventilation (MV) + TTDN100% group had the lowest lung-volume loss over the study (*n* = 6 for all groups, mixed-methods analysis). *B*: change in static compliance over time was significantly affected by TTDN, and the MV + TTDN100% group demonstrated best overall compliance throughout the study (*n* = 6 for all groups, mixed-methods analysis). *C*: change in transpulmonary plateau pressure over time was significantly affected by TTDN, and the MV + TTDN100% group had the lowest overall pressure throughout the study (*n* = 6 for all groups, mixed-methods analysis).

The change in end-expiratory lung volume (EELV), normalized to weight, showed a trend to significantly lower volume lost in MV + TTDN100% postinjury (*P* = 0.058, Table 2). Study-end EELV loss was lowest in MV + TTDN100% (*P* = 0.007, Table 2). The change in EELV over time was only affected by time (*P* < 0.001, Table 2, Fig. 2*A*).

Static compliance was significantly different and highest in MV + TTDN100% at baseline (*P* = 0.041), postinjury (*P* = 0.006), and study-end (*P* = 0.014). Refer to Table 3: static compliance over time was significantly affected by time (*P* < 0.001) and TTDN dose (*P* = 0.010), but not the interaction of TTDN dose and time (Table 3, Fig. 2*B*).

**Table 3.** Static lung compliance, transpulmonary plateau pressure, transpulmonary driving pressure, and mechanical power measured at baseline, postinjury, and study-end

	Median (IQR)			Kruskal–Wallis (*P* Value)	Dunn’s Multiple Comparisons Post Hoc Test		Mixed Methods Analysis (*P* Value)		
	MV (*n* = 6)	MV + TTDN50% (*n* = 6)	MV + TTDN100% (*n* = 6)		Groups	*P* Value	Time	TTDN	TTDN × Time
Static lung compliance, mL/cmH_2_O									
Baseline	63 (43–77)	74 (51–79)	94 (80–99)	**0.041**	MV vs. MV + TTDN50%	ns	**<0.001**	**0.010**	ns
					MV vs. MV + TTDN100%	ns (0.059)			
					MV + TTDN50% vs. MV + TTDN100%	ns			
Injury	32 (28–49)	41 (33–67)	45 (43–55)	**0.006**	MV vs. MV + TTDN50%	**0.047**			
					MV vs. MV + TTDN100%	**0.010**			
					MV + TTDN50% vs. MV + TTDN100%	ns			
Study-end	33 (28–38)	39 (35–60)	59 (40–79)	**0.014**	MV vs. MV + TTDN50%	ns			
					MV vs. MV + TTDN100%	**0.018**			
					MV + TTDN50% vs. MV + TTDN100%	ns			
Transpulmonary plateau pressure, cmH_2_O									
Baseline	5.5 (3.6–6.3)	6.0 (4.5–6.1)	4.8 (4.3–5.3)	ns	MV vs. MV + TTDN50%		**<0.001**	**0.005**	ns
					MV vs. MV + TTDN100%				
					MV + TTDN50% vs. MV + TTDN100%				
Injury	12.0 (9.1–14.5)	10.0 (6.8–10.6)	7.9 (4.6–8.9)	ns (0.055)	MV vs. MV + TTDN50%				
					MV vs. MV + TTDN100%				
					MV + TTDN50% vs. MV + TTDN100%				
Study-end	13.1 (12.2–15.6)	11.5 (8.1–14.5)	8.1 (5.4–11.1)	ns (0.053)	MV vs. MV + TTDN50%				
					MV vs. MV + TTDN100%				
					MV + TTDN50% vs. MV + TTDN100%				
Transpulmonary driving pressure, cmH_2_O									
Baseline	8.2 (5.7–8.9)	7.6 (6.2–9.1)	5.2 (4.7–6.1)	**0.040**	MV vs. MV + TTDN50%	ns	**<0.001**	**<0.001**	ns
					MV vs. MV + TTDN100%	ns			
					MV + TTDN50% vs. MV + TTDN100%	ns			
Injury	14.5 (11.6–17.1)	10.8 (9.1–12.5)	9.8 (8.3–10.9)	**0.024**	MV vs. MV + TTDN50%	ns			
					MV vs. MV + TTDN100%	**0.033**			
					MV + TTDN50% vs. MV + TTDN100%	ns			
Study-end	14.8 (13.2–16.5)	11.4 (9.9–13.8)	8.6 (6.2–11.9)	**0.006**	MV vs. MV + TTDN50%	ns			
					MV vs. MV + TTDN100%	**0.008**			
					MV + TTDN50% vs. MV + TTDN100%	ns			
Mechanical power, J/min									
Baseline	15 (14–19)	14 (12–20)	15 (14–15)	ns	MV vs. MV + TTDN50%		**0.002**	**0.041**	**0.011**
					MV vs. MV + TTDN100%				
					MV + TTDN50% vs. MV + TTDN100%				
Injury	20 (17–25)	22 (20–28)	17 (14–21)	ns	MV vs. MV + TTDN50%				
					MV vs. MV + TTDN100%				
					MV + TTDN50% vs. MV + TTDN100%				
Study-end	23 (19–27)	22 (18–24)	16 (14–20)	**0.028**	MV vs. MV + TTDN50%	ns			
					MV vs. MV + TTDN100%	**0.045**			
					MV + TTDN50% vs. MV + TTDN100%	ns			

Change over time was compared using mixed-methods analysis. MV, mechanical ventilation; ns, not significant; TTDN, temporary transvenous diaphragm neurostimulation. Bold values are significant *P* values.

Transpulmonary plateau pressure was not different between groups at baseline and showed a trend to significantly lower in MV + TTDN100% postinjury (*P* = 0.055, Table 3). Study-end measurements also showed a trend to significantly lower in MV + TTDN100% (*P* = 0.053, Table 3). Transpulmonary plateau pressure over time was significantly affected by time (*P* < 0.001) and TTDN dose (*P* = 0.005), whereas the interaction of TTDN dose and time was not significant (Table 3, Fig. 2*C*).

### Transpulmonary Driving Pressure, Mechanical Power, and Mechanical Work

**Figure 3.**
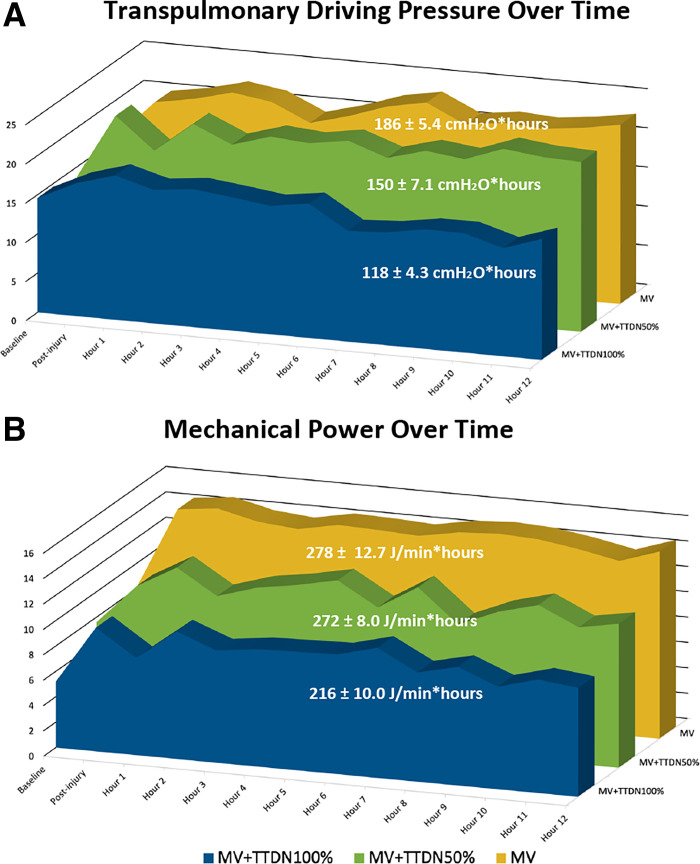
*A*: total study exposure to transpulmonary driving pressure over time, as measured by area-under-the-curve, was significantly lowest in the mechanical ventilation (MV) + temporary transvenous diaphragm neurostimulation (TTDN)100% group (*n* = 6 in all groups, ANOVA). *B*: total study exposure to mechanical power over time, as measured by area-under-the-curve, was significantly lowest in the MV + TTDN100% group (*n* = 6 in all groups, ANOVA).

Transpulmonary driving pressure was significantly different and lowest in MV + TTDN100% at baseline (*P* = 0.040), postinjury (*P* = 0.024), and study-end (*P* = 0.024). Refer to Table 3: transpulmonary driving pressure over time was significantly affected by time (*P* < 0.001), and TTDN dose (*P* < 0.001), and the interaction of TTDN dose and time showed a trend to significance (*P* = 0.067, Table 3). Total study exposure (area under the curve) to transpulmonary driving pressure was lowest in MV + TTDN100% (*P* < 0.001, Table 4, Fig. 3*A*). Study-end transpulmonary driving pressure had a trend to moderate correlation with study-end EELV loss (*r* = 0.4636, *P* = 0.053).

**Table 4.** Total study exposure to mechanical power, mechanical work, and driving pressure as measured by total area under the curve

	MV (*n* = 6)	MV + TTDN50% (*n* = 6)	MV + TTDN100% (*n* = 6)	ANOVA (*P* Value)
Transpulmonary driving pressure, (cmH_2_O) × h				
Total area under the curve	186	150	118	**<0.001**
Std. error	5.4	7.1	4.3	
95% Confidence interval	175–196	137–164	110–127	
% Difference from MV		−19%	−36%	
Mechanical power, (J/min) × h				
Total area under the curve	278	272	216	**<0.001**
Std. error	12.7	8.0	10.0	
95% Confidence interval	254–303	256–287	197–235	
% Difference from MV		−2%	−22%	
Total mechanical work (system PTP per breath) [positive (ventilator) + negative (Diaphragm) pressure], (cmH_2_O·s) × h				
Total area under the curve	129	120	104	**0.001**
Std. error	5.3	3.2	2.8	
95% Confidence interval	119–139	114–126	99–110	
% Difference from MV		−7%	−19%	
Elastic mechanical work (Nonintrinsic PEEP elastic PTP per breath), (cmH_2_O·s) × h				
Total area under the curve	76	70	56	**0.009**
Std. error	3.7	5.2	2.0	
95% Confidence interval	68–83	59–80	53–60	
% Difference from MV		−8%	−26%	
Resistive mechanical work (resistive PTP per breath), (cmH_2_O·s) × h				
Total area under the curve	44	47	44	ns
Std. error	3.6	3.8	2.2	
95% Confidence interval	37–51	40–55	40–48	
% Difference from MV		+9%	0%	

MV, mechanical ventilation; TTDN, temporary transvenous diaphragm neurostimulation. Bold values are significant *P* values.

Mechanical power was not significantly different at baseline. MV + TTDN100% required the lowest mechanical power for adequate ventilation postinjury (*P* = 0.016) and at study-end (*P* = 0.029). Refer to Table 3: mechanical power over time was affected by time (*P* < 0.001), TTDN dose (*P* = 0.041), and the interaction of TTDN dose and time (*P* = 0.011, Table 3). Total study mechanical power exposure (area under the curve) was lowest in MV + TTDN100% (*P* < 0.001, Table 4, Fig. 3*B*). Study-end mechanical power was moderately-to-strongly correlated with study-end EELV loss (*r* = 0.6656, *P* = 0.003) and moderately correlated to lung injury scores (*r* = 0.4985, *P* = 0.035).

**Figure 4.**
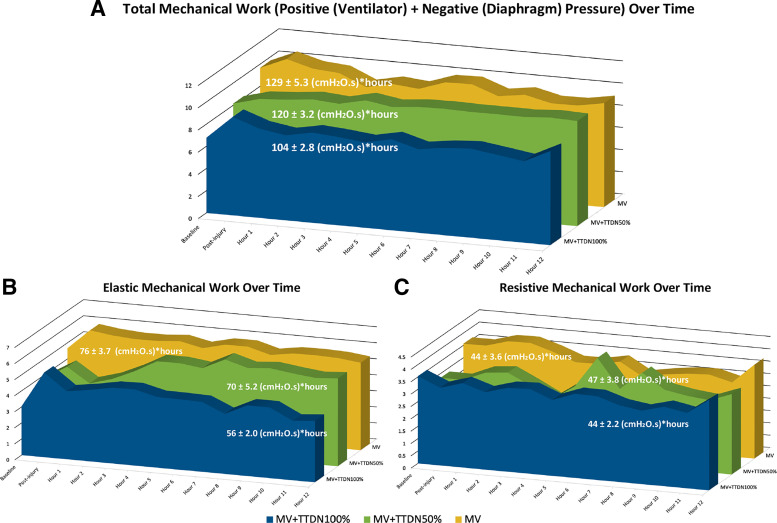
*A*: total mechanical work over time, as measured by area-under-the-curve, was significantly lowest in the mechanical ventilation (MV) + temporary transvenous diaphragm neurostimulation (TTDN)100% group (*n* = 6 in all groups, ANOVA). *B*: elastic mechanical work over time, as measured by area-under-the-curve, was significantly lowest in the MV + TTDN100% group (*n* = 6 in all groups, ANOVA). *C*: resistive mechanical work over time, as measured by area-under-the-curve, was not different between groups (*n* = 6 in all groups, ANOVA).

Total study exposure, as measured by area under the curve, to mechanical work due to both positive pressure (ventilator PTP) plus negative pressure (diaphragm PTP) was lowest for MV + TTDN100% (*P* = 0.001, Table 4, Fig. 4*A*). Total study exposure to elastic mechanical work (nonintrinsic PEEP elastic PTP) was lowest for MV + TTDN100% (*P* = 0.009, Table 4, Fig. 4*B*). Total study exposure to resistive mechanical work (resistive PTP) was not significantly different for all groups (Table 4, Fig. 4*C*). Refer to the Supplemental Material for detailed mechanical work data.

### Alveolar Chord Length

**Figure 5.**
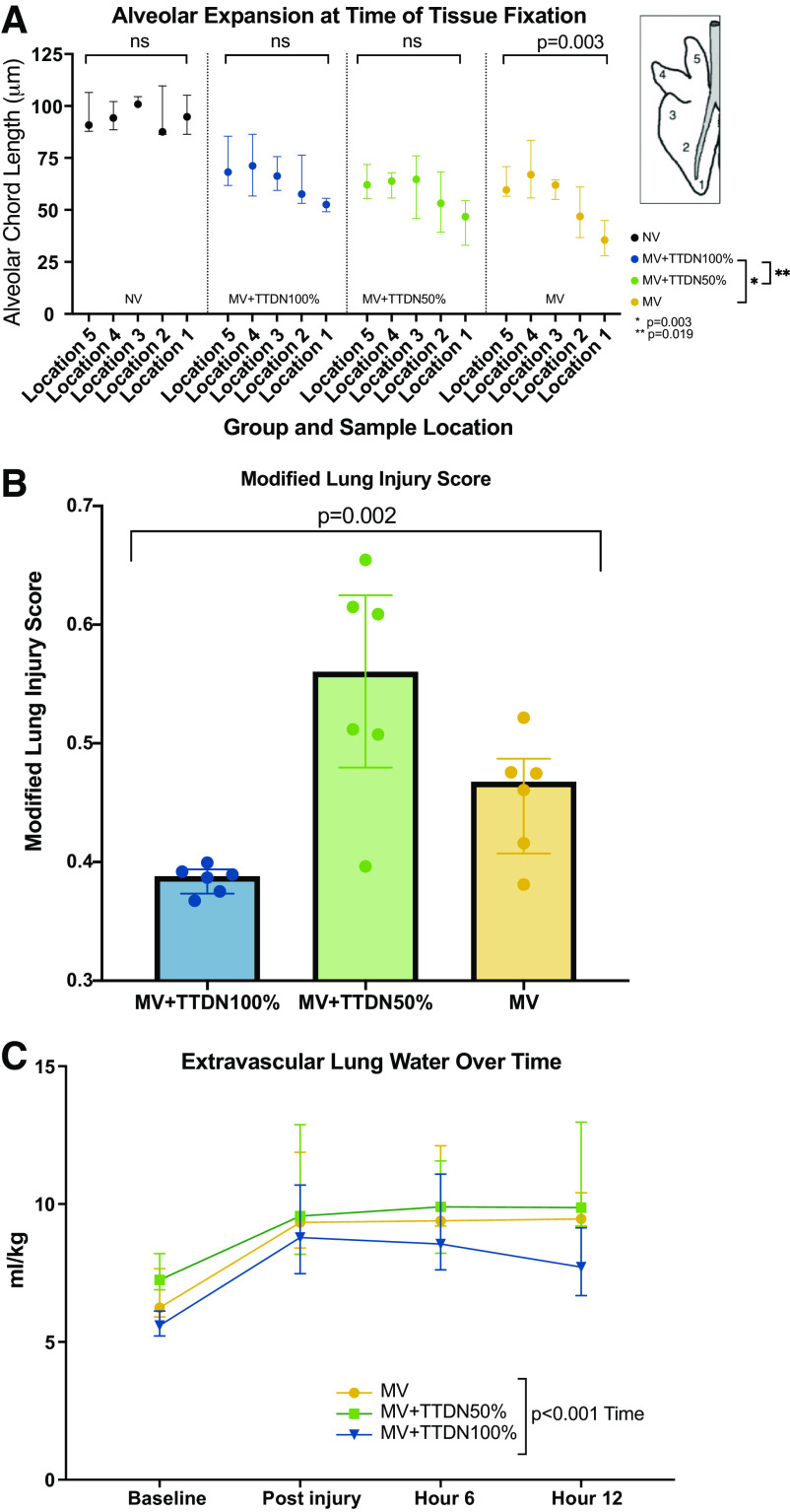
*A*: alveolar expansion at time of tissue fixation, showing a significant difference between the mechanical ventilation (MV) + temporary transvenous diaphragm neurostimulation (TTDN)100% group compared with both the MV group and the MV + TTDN50% group (*n* = 6 for all groups, Kruskal–Wallis analysis with Dunn’s multiple comparison test post hoc). *B*: lung injury score was significantly different and lowest in the MV + TTDN100% group (*n* = 6 for all groups, Kruskal–Wallis analysis). *C*: extravascular lung water over time was lowest in the MV + TTDN100% group, but only significantly affected by time (*n* = 6 for all groups, mixed-methods analysis).

Alveolar chord length was not significantly different between sample locations in the NV group, the MV + TTDN100% group, and the MV + TTDN50% group. Alveolar chord length was significantly different between sample locations in the MV group (*P* = 0.003, Table 5, Fig. 5*A*).

**Table 5.** Alveolar expansion at time of tissue fixation (alveolar chord length)

Location	Units	Median (IQR)			
		MV (*n* = 6)	MV + TTDN50% (*n* = 6)	MV + TTDN100% (*n* = 6)	NV (*n* = 6)
Sample location 5	µm	60 (57–71	62 (56–72)	68 (62–86)	91 (88–107)
Sample location 4	µm	67 (56–84)	64 (56–68)	71 (57–86)	94 (89–102
Sample location 3	µm	62 (55–64)	65 (46–76)	66 (59–76)	101 (99–105)
Sample location 2	µm	47 (37–61)	53 (39–68)	58 (53–76)	88 (86–110)
Sample location 1	µm	36 (28–45)	47 (33–54)	53 (49–56)	95 (86–105)
Kruskal–Wallis (*P* value)		0.003	ns	ns	ns
Dunn’s Multiple Comparisons (post-hoc) (*P* value)	5 vs. 4	ns			
	5 vs. 3	ns			
	5 vs. 2	ns			
	5 vs. 1	0.032			
	4 vs. 3	ns			
	4 vs. 2	0.023			
	4 vs. 1	0.005			
	3 vs. 2	ns			
	3 vs. 1	0.031			
	2 vs. 1	ns			

MV, mechanical ventilation; ns, not significant; NV, never-ventilated; TTDN, temporary transvenous diaphragm neurostimulation.

### Modified Lung Injury Score

**Figure 6.**
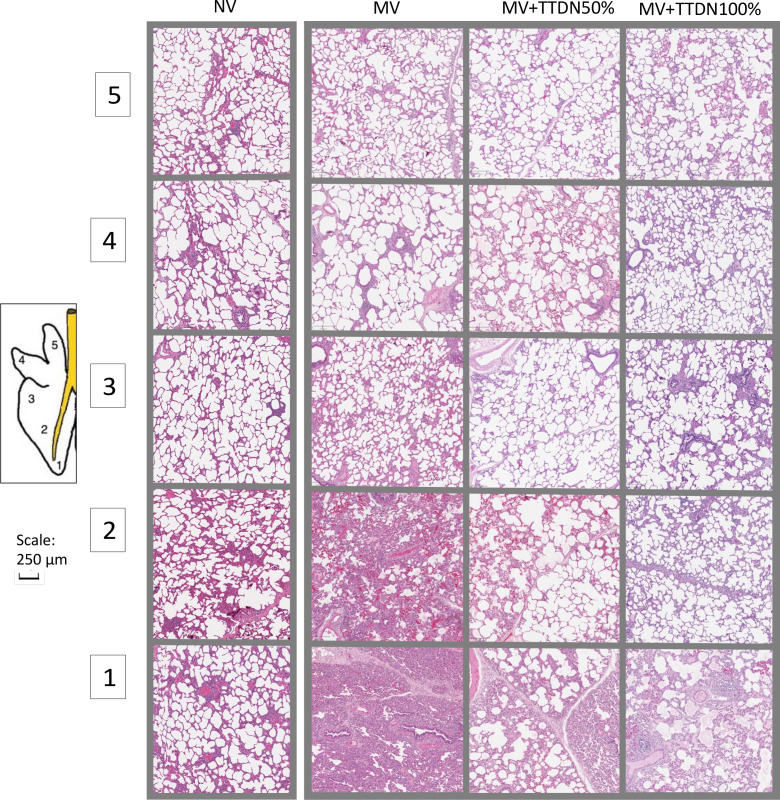
Photomicrographs of representative samples of hematoxylin-eosin (H&E)-stained lung tissue at each of the five sampled locations, showing differences in tissue morphology and patterns of alveolar expansion between the groups. MV, mechanical ventilation; NV, never-ventilated; TTDN, temporary transvenous diaphragm neurostimulation.

The modified lung injury score was significantly different between groups and lowest in MV + TTDN100% (*P* = 0.002, Table 1, Fig. 5*B*). See Fig. 6 for representative lung tissue photomicrographs of all sample regions in all groups.

### Extravascular Lung Water

Extravascular lung water (EVLW), normalized to weight, was highest in MV + TTDN50% at baseline (*P* = 0.010, Table 2), and lowest in MV + TTDN100% at study-end (*P* = 0.027, Table 2). EVLW over time was only affected by time (*P* < 0.001, Table 2, Fig. 5*C*).

### Lung Tissue and Serum Cytokines

Total lung tissue cytokine concentration (the sum of all 13 cytokine concentration measurements) was significantly different between the three ventilated groups (*P* = 0.008) with the lowest total concentration measured in the MV + TTDN100% group: 10,372.4 pg/mL MV versus 9,394.7 pg/mL MV + TTDN50% versus 6,887.5 pg/mL MV + TTDN100%. Post hoc analysis identified that MV versus MV + TTDN100% was different (*P* = 0.017) and MV + TTDN50% versus MV + TTDN100% was different (*P* = 0.021). Detailed values for all cytokines measured are reported in the Supplemental Material. Total serum cytokine concentration profile was not significantly different and detailed data for all cytokines measured are reported in the Supplemental Material.

### Adverse Events, Ventilator Asynchrony and Spontaneous Efforts

No adverse events or dangerous arrhythmias were observed during the study. No significant ventilator asynchrony (breath stacking/reverse triggering) was observed. Spontaneous efforts were a median of 6–10 single efforts per total study period and not significantly different between groups.

## DISCUSSION

Twelve hours of volume-control mechanical ventilation plus TTDN on every breath, termed negative-pressure-assisted ventilation, resulted in improved $\text{P}{\text{a}}_{{\text{O}}_{2}}$/$\text{F}{\text{I}}_{{\text{O}}_{2}}$, A-a gradient, and alveolar homogeneity, as well as reduced driving pressure, mechanical power, lung injury score, and lung tissue cytokines post-oleic acid-induced lung injury, when compared with volume-control ventilation alone. This study demonstrates that TTDN is a promising ventilation adjunct that can improve protective lung ventilation for patients with ARDS and is appropriate to investigate in humans.

MV + TTDN100% resulted in the highest $\text{P}{\text{a}}_{{\text{O}}_{2}}$/$\text{F}{\text{I}}_{{\text{O}}_{2}}$ at study-end, with MV + TTDN50% being similar to MV, suggesting that TTDN dose may be important. This study’s ventilation protocol used a tidal volume within the ARDSNet lung-protection recommendation, thus substantial VILI was likely mitigated, allowing for functional recovery from the initial oleic acid-induced injury in all groups (5). However, recovery was greatest in MV + TTDN100%. We previously published that MV + TTDN100%, using this same protocol, mitigated $\text{P}{\text{a}}_{{\text{O}}_{2}}$/$\text{F}{\text{I}}_{{\text{O}}_{2}}$ reduction in healthy-lung pigs ventilated for 50 h, indicating that TTDN improves $\text{P}{\text{a}}_{{\text{O}}_{2}}$/$\text{F}{\text{I}}_{{\text{O}}_{2}}$ in both homogeneous and heterogeneous lungs (23). As $\text{P}{\text{a}}_{{\text{O}}_{2}}$/$\text{F}{\text{I}}_{{\text{O}}_{2}}$ has several limitations, A-a gradient is also reported for this study (34, 35). All groups had a similarly elevated A-a gradient postinjury, however MV + TTDN100% had a better recovery at study-end, indicating that pulmonary shunt was less persistent in this group at study-end. MV + TTDN100% restored lung function and reduced intrapulmonary shunt the most and is likely the more beneficial lung-protection strategy.

Driving pressure and mechanical power are both independently associated with increased mortality in ventilated patients (5, 7, 9, 36, 37). Transpulmonary driving pressure was the lowest postinjury and at study-end in MV + TTDN100%. The total study exposure to transpulmonary driving pressure was 36% lower in MV + TTDN100% compared with MV, reducing the risk for VILI in this group. Mauri et al. (38) recommend transpulmonary driving pressure should be limited to < 10–12 cmH_2_O to limit VILI in patients with inhomogeneous lung parenchyma. MV + TTDN100% limited transpulmonary driving pressure below 10 cmH_2_O postinjury and at study-end. MV + TTDN50% limited transpulmonary driving pressure below 12 cmH_2_O postinjury and at study-end. Amato et al. (10) have reported increased mortality with a relative risk of death of 1.41 when there is an increase in driving pressure of ∼7 cmH_2_O. Transpulmonary driving pressure was 6.2 cmH_2_O higher in MV versus MV + TTDN100% at study-end. Mechanical power was also the lowest in MV + TTDN100% postinjury and at study-end. The total study exposure to mechanical power was 22% less for MV + TTDN100% compared with MV. Neto et al. (37) reported a consistent increase in risk of death in humans when mechanical power was >17.0 J/min for the first 48 h, and increased in-hospital mortality for every 5 J/min increase. MV + TTDN100% required mechanical power <17 J/min at study-end and was 7 J/min lower than MV. These data demonstrate that MV + TTDN100% delivered safe transpulmonary driving pressure and mechanical power during the postinjury ventilation period, which is further supported by the moderate correlation between mechanical power and modified lung injury scores. Not only does TTDN offer a new way to reduce driving pressure and mechanical power during ventilation, but it should also be investigated in humans using an “every breath” strategy. If these results translate to human patients with ARDS, then negative-pressure-assisted ventilation has the potential to further reduce the relative risk of death in sedated patients with ARDS receiving lung-protective mechanical ventilation.

TTDN mitigated EELV loss effectively in the MV + TTDN100% group. We previously published that TTDN restores a more physiologically normal distribution of tidal volume, reducing alveolar collapse that drives the loss of EELV (23). Interestingly, a similar amount of atelectasis was measured in this study as was measured after 50 h in our previous healthy-lung study (23). This highlights how ARDS accelerates atelectasis and promotes regional alveolar collapse leading to lung heterogeneity compared with a noninjured lung (27, 39, 40). The correlation between EELV loss and mechanical power in this study further highlights the link of atelectasis formation to risk factors for VILI. Static compliance was also the highest in MV + TTDN100%, at all timepoints, reflecting improved lung tissue elastance. Not only is this likely due to improved tidal-volume distribution and preserved EELV, but we also hypothesize that MV + TTDN100% restores diaphragm tone during expiration, combating abdominal hydrostatic pressure and further improving compliance (14, 41). During normal spontaneous respiration, the diaphragm maintains tone throughout the expiratory cycle, thereby preserving EELV and we hypothesize that MV + TTDN100% restores this residual tone (14, 41). This suggests that MV + TTDN100% may be suitable for future lung-protection protocols, and its influence on diaphragm tone is an area for future studies.

PTP is a measure of the mechanical work of breathing and describes the work that needs to be performed by either the ventilator or the patient to deliver volume to the alveoli (42). The strength of TTDN diaphragm contractions in our study (negative-pressure, or diaphragm, PTP) specifically targeted a 15%–20% reduction of the work performed by the ventilator (positive-pressure, or ventilator, PTP). Combining both ventilator mechanical work and diaphragm mechanical work describes the total burden of mechanical work required to support the animal for the duration of the study. The total study mechanical work (ventilator + diaphragm PTP) was 19% lower in MV + TTDN100% compared with MV, demonstrating that TTDN may reduce the total work required to sustain critically ill patients, reducing their exposure risk for VILI. This reduction was due to 26% reduced elastic mechanical work and is further supported by improved static compliance, preserved EELV, and reduced driving pressure seen in MV + TTDN100%.

Alveolar chord length is a measure of alveolar expansion at the time of tissue fixation and can be used to describe alveolar homogeneity (32). Although all groups demonstrated smaller alveolar chord lengths than the true-control NV group, both MV + TTDN50% and MV + TTDN100% showed no significant difference between sample locations. This supports that TTDN preserved alveolar homogeneity in this ARDS model. We previously published that TTDN on every breath preserved alveolar homogeneity in healthy-lung pigs ventilated for 50 h (23). This supports that TTDN helps maintain alveolar homogeneity in both a heterogeneous and homogeneous lung and offers a novel way of combating regional atelectasis in ventilated human patients.

Lung injury scores were lowest in MV + TTDN100%, suggesting mitigated lung injury in this group. The higher score in MV + TTDN50% is an unexpected finding and may be related to the alternating pattern and stress profile of a TTDN-breath followed by a passive-diaphragm breath. Perhaps this is because the driving pressure and mechanical power are only reduced on half the breaths and the distribution of ventilation is changing breath-to-breath. MV + TTDN50% also did not have a significant effect on EELV loss. We recommend that future human studies evaluating TTDN to protect the lungs in humans be provided on every breath.

MV + TTDN100% had the lowest overall total lung tissue cytokines. This suggests that this group had a lower inflammatory reaction despite receiving the same lung injury and ventilation protocol as the other groups. This is supported by the improved $\text{P}{\text{a}}_{{\text{O}}_{2}}$/$\text{F}{\text{I}}_{{\text{O}}_{2}}$ and A-a gradient, as well as lower lung injury scores in this group. As this study is underpowered for this outcome and the specific impact of individual cytokines and their values are not well elucidated in pigs, we have reported total tissue cytokine concentrations in this manuscript and individual detailed analysis can be found in the Supplemental Material.

EVLW over time is associated with mortality in ARDS (43, 44). EVLW, indexed to weight, increased from baseline and was ≤10 mL/kg for all groups (considered normal) at study-end (45). However, EVLW was lowest in MV + TTDN100% at study-end, indicating that this group demonstrated a partial reduction of EVLW over the study, whereas the other groups maintained slightly elevated EVLW. TTDN on every breath either produced less EVLW or increased clearance of EVLW over the study. Less EVLW reduces the risk for alveolar collapse through surfactant alteration and improves gas exchange as less alveoli are flooded. MV + TTDN100% did not worsen pulmonary edema through diaphragm-contracted transpulmonary pressure swings. This is relevant as in critically ill patients there is often a requirement for fluid resuscitation that may result in increased EVLW, whereas in our study we strictly controlled fluid administered. Thus, the effects of MV + TTDN100%’s protective effect on EVLW may be more important in patients who require additional fluid.

Our study is limited by the use of a preclinical ARDS model with a small number of subjects. This oleic-acid injury model produces acute and repair phases with histopathological and physiological features similar to human ARDS, however, it does not model the physiopathology of septic ARDS (29, 46, 47). This model meets the new American Thoracic Society recommendations for animal models of lung injury as it contains histological evidence of tissue injury, alteration of the alveolar-capillary barrier, presence of an inflammatory response, and evidence of physiological dysfunction (48). The goal of this study was to investigate the effects that TTDN has on ventilator-induced lung injury, the results of which may apply to heavily sedated, critically ill patients receiving controlled mechanical ventilation. Whether the results are the same in human ICU subjects with ARDS remains to be proven. ICU patients at risk for VILI are typically ventilated for longer than this study, however, a longer duration of this experiment would have been technically difficult.

This study builds upon our research in a healthy-lung preclinical model, describing how TTDN also offers protection against VILI in an ARDS model. Our other research details how TTDN mitigates diaphragm atrophy and ventilator-associated brain injury (23, 24, 26). Negative-pressure-assisted ventilation using TTDN offers promise as a single-technology solution to the ICU dilemma of providing diaphragm, lung, and brain protection simultaneously to critically ill patients.

### Conclusions

Twelve hours of volume-control ventilation combined with TTDN on every breath resulted in improved $\text{P}{\text{a}}_{{\text{O}}_{2}}$/$\text{F}{\text{I}}_{{\text{O}}_{2}}$, A-a gradient, and alveolar homogeneity, and reduced driving pressure, mechanical power, lung injury score, and tissue cytokine concentration post-oleic acid-induced lung injury, compared with volume-control ventilation alone. This exciting technology offers a new way to protect the lungs of critically ill, ventilated patients.

## SUPPLEMENTAL DATA

10.6084/m9.figshare.20514132 Supplemental Material: https://doi.org/10.6084/m9.figshare.20514132.

## GRANTS

This study was supported by Lungpacer Medical USA, Inc., Royal Columbian Hospital Foundation, TB Vets Foundation, and BC Lung Association.

## DISCLOSURES

Matt Gani and Thiago Bassi are employees of Lungpacer Medical Inc. Steven Reynolds is an investor in Lungpacer Medical Inc. All other authors have received consulting fees from Lungpacer Medical Inc.

## AUTHOR CONTRIBUTIONS

E.C.R., M.G., and S.C.R. conceived and designed research; E.C.R., T.G.B., M.N., J.W., M.O., and K.C.F. performed experiments; E.C.R., T.G.B., M.N., and J.W. analyzed data; E.C.R., T.G.B., M.N., M.G., and S.C.R. interpreted results of experiments; E.C.R. prepared figures; E.C.R. drafted manuscript; E.C.R., M.G., and S.C.R. edited and revised manuscript; E.C.R., T.G.B., M.N., J.W., M.O., K.C.F., M.G., and S.C.R. approved final version of manuscript.
